# PCR-denaturing Gradient Gel Electrophoresis as a Simple Identification Tool of Arbuscular Mycorrhizal Fungal Isolates

**DOI:** 10.1264/jsme2.ME19074

**Published:** 2019-12-27

**Authors:** Ryo Ohtomo, Norikuni Oka, Sho Morimoto

**Affiliations:** 1 Central Region Agricultural Research Center, NARO 2–1–18 Kannondai, Tsukuba, Ibaraki 305–8666 Japan; 2 Hokkaido Agricultural Research Center, NARO 1 Hitsujigaoka, Toyohira, Sapporo, Hokkaido 062–8555 Japan

**Keywords:** arbuscular mycorrhizal fungi, culture collection, PCR-denaturing gradient gel electrophoresis, quality control

## Abstract

Due to their obligate symbiotic nature and lack of long-term storage methods, the strain collection of arbuscular mycorrhizal (AM) fungi requires periodic proliferation using a pot culture with host plants. Therefore, a method to evaluate the purity of proliferated AM fungal cultures is critical for the quality control of their collection. In a simple evaluation of the purity and identity of a proliferated AM fungal culture, DNA extracted from the culture was amplified using AM fungi-specific PCR followed by an analysis with denaturing gradient gel electrophoresis (PCR-DGGE). The present results showed that the DGGE band patterns of AM fungal strains differed according to their phylogenetic positions, allowing for the rapid and easy identification of the proliferated AM fungal strains. When a culture was contaminated with another AM fungal strain, the DGGE pattern became a mixture of those strains. A contaminant strain was detectable even when its ratio was 1/9 of the main strain. It was also possible to confirm the purity of the culture by comparing whether the DGGE band pattern of the proliferated culture was identical to that obtained from single spores isolated from the culture. Therefore, PCR-DGGE is useful as a quality control tool for maintaining culture collections of AM fungi.

Arbuscular mycorrhiza (AM) is one of the most common plant-microbe interactions in the terrestrial ecosystem. More than 70% of land plants form symbiotic interactions with AM fungi, and this symbiosis has wide-ranging functions (34), including enhancing nutrient uptake (particularly phosphate) by host plants (33, 35), alleviating environmental stresses, such as drought (13) or heavy metal pollution of soil (8, 11, 43), and mitigating damages caused by pests and insects (1, 42). The symbiosis also affects other plant-microbe interactions, such as legume–rhizobia symbiosis (2, 7, 40), and plays important roles in environmental protection, such as in the stabilization of soil aggregates (25, 29) and reducing soil nutrient loss (5).

As of February 2019, more than 300 fungal species have been listed as AM fungi (http://www.amf-phylogeny.com/index.html) and belong to Glomeromycota (32) (although a reclassification of the group to the subphylum Glomeromycotina under the phylum Mucoromycota was recently proposed [36], the phylum Glomeromycota remains valid [39]). These species’ functions and life histories differ according to the strain (6). To obtain a deeper understanding of their biology and function and to more effectively utilize AM functions in ecosystem preservation and agricultural production, it is necessary to clarify the specific features of each AM fungus. Therefore, several culture collections of AM fungi have been established worldwide (3). In Japan, the National Agricultural Research Organization (NARO) Genebank project (https://www.gene.affrc.go.jp/index_en.php) maintains pure cultures of several AM fungal strains and distributes them for research purposes upon request.

Due to their obligate symbiotic nature and lack of long-term storage methods (17), a periodic pot culture of AM fungi is required to maintain their cultures. However, after every pot culture, it is necessary to confirm whether only target fungal species have proliferated. The identification of AM fungal strains and examination of their purity in cultures have traditionally relied on spore morphology (4), which requires technical proficiency and time-consuming labor (15). Molecular biological information, such as rRNA gene sequences, has recently become the main marker for identifying fungal strains.

PCR-denaturing gradient gel electrophoresis (PCR-DGGE) is a method that separates DNA fragments based on sequence differences and has been used in the field of molecular ecology since approximately 2000 (37). Previous studies reported the use of PCR-DGGE to identify AM fungi or elucidate AM fungal compositions in the environment (10, 18, 19, 22, 24, 31). In the present study, we applied PCR-DGGE to simplify the identification of isolated AM fungi and check the purity of proliferated AM fungal strains. Our aim was to assess the usefulness of this method as a quality control tool for AM fungal culture collection.

## Materials and Methods

### AM fungal strains

**Fig. 1:**
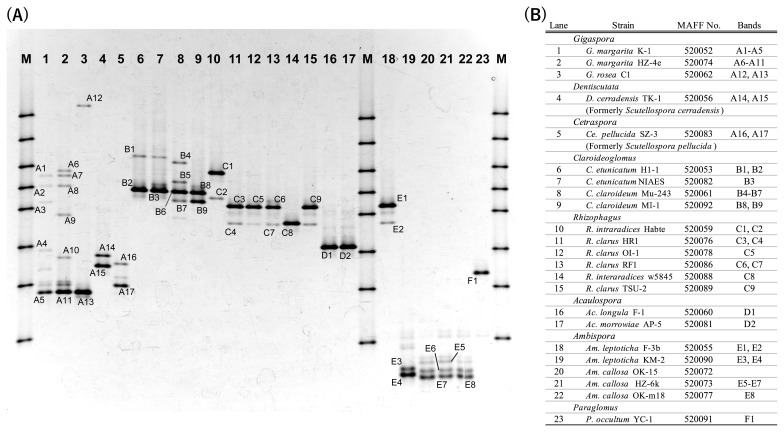
PCR-DGGE patterns of AM fungal cultures. (A) Amplicons of AM fungi-specific PCR of each AM fungal culture were loaded onto a DGGE gel. DGGE bands with nucleotide sequences that were analyzed are marked with letters+numbers. M, marker lane (DGGE Marker IV, Nippon Gene). (B) List of cultures analyzed in A.

All AM fungal strains used in the present study were obtained from the NARO Genebank. The strains used in the present study other than those listed in Fig. 1B were *Gigaspora margarita* Ni-A (MAFF140115) and *Gi. margarita* C (MAFF520054). Each strain was propagated through 4–6 months of a pot culture using bahia grass (*Paspalum notatum* Fluegge) and white clover (*Trifolium repens* L.) as hosts, and a mixture of sterile black soil, river sand, and akadama soil in a volume ratio of 1:1:1 as media. After fungal proliferation, the pots were naturally air dried for one month; pot media were passed through a 2-mm sieve to remove large plant material and then stored at 4°C as proliferated AM fungal cultures.

AM fungal cultures that were propagated in 2016 were used for the comparison of DGGE patterns of different AM fungal strains. *Gi. margarita* spores used to compare spore DGGE patterns were isolated from 2015 cultures. AM fungal cultures propagated in 2017 were used in the DGGE analysis of contaminated templates.

### DNA extraction from soil or spores

DNA from AM fungal cultures (*i.e.*, a soil-sand mixture containing AM fungal spores, hyphae, and small root pieces of host plants) were extracted using the FastDNA SPIN Kit for Soil (MP Bio, Solon, OH, USA) and further purified with the DNA Clean & Concentrator-25 kit (Zymo Research, Orange, CA, USA) following the providers’ instructions. We generally started with 0.4 g of soil samples and ended with a final elution volume of 30 μL. Eighty microliters of 20% steam-sterilized skim milk (Becton, Dickinson and Company, Franklin Lakes, NJ, USA) was included in the first step of the extraction protocol to improve DNA recovery (38).

To prepare DNA from a single spore, spores were recovered from AM fungal cultures using a wet sieving method, followed by sucrose density gradient centrifugation and then picking up spores using forceps (4). Isolated spores were washed once in 0.05% (w/v) sterile Tween 20 and once in sterile distilled water with sonication. Regarding large spores of *Gigaspora*, 20 μL of well mixed InstaGene matrix (Bio-Rad Laboratories, Hercules, CA, USA) was added to a single spore in a 0.2-mL thin-walled PCR tube. The spore was crushed thoroughly using a long 10-μL pipette tip with a heat sealed point. Using a thermal cycler, the tubes were heated at 56°C for 30 min, then at 95°C for 10 min, followed by cooling at 4°C. The tubes were then spun, and the supernatant was recovered and used directly as a PCR template. Regarding small spores of *Claroideoglomus*, 3 μL of TE buffer (pH 8.0) was used instead of the InstaGene matrix, and the extract was prepared using the same procedure as described above.

### PCR amplification and DGGE analysis

An AM fungi-specific PCR-DGGE analysis was performed as described by Morimoto *et al.* (24). Briefly, 1 μL of template DNA was amplified using primers targeting the AM fungal 18S rRNA genes, GC-AMV4.5NF (forward primer, [31]) and AMVR (reverse primer, [24]). PCR was performed in a 30-μL volume comprising 1× KOD-Plus buffer, 0.2 mM of each dNTP, 1.0 mM MgSO_4_, 0.6 μL of 20 mg mL^−1^ bovine serum albumin solution (TAKARA Bio, Kusatsu, Japan), 0.2 μM of each primer, 0.6 U KOD-Plus DNA polymerase (TOYOBO, Osaka, Japan), and 1 μL of template DNA. PCR conditions were as follows: initial denaturation at 94°C for 2 min followed by 45 cycles of 94°C for 15 s, 56°C for 30 s, and 68°C for 30 s. The primer set showed lower amplification efficiency for the 18S rRNA of *Ambispora* and *Paraglomus*. Therefore, soil DNA was pre-amplified using the same conditions as described above, except that the forward primer without a GC-clamp (AMV4.5NF) was added and only 20 cycles were used to perform amplification on cultures of these strains. After 20 cycles, 1 μL of the reaction mixture was used as a template in second PCR consisting of 25 amplification cycles and a reaction mixture using a forward primer with a GC-clamp. Amplified DNA (*ca.* 320 bp) were purified using the QIAquick PCR Purification Kit (Qiagen, Hilden, Germany) and then quantified using a NanoDrop ND-2000 spectrophotometer (ThermoFisher Scientific, Waltham, MA, USA).

Forty nanograms of each PCR product was loaded onto an 8% polyacrylamide gel with a 25–40% denaturing gradient and electrophoresed at 50 V for 16 h at 60°C using a DCode universal mutation detection system (Bio-Rad Laboratories) as described by Morimoto *et al.* (24). Six microliters of DGGE Marker IV (8 fragments, Nippon Gene, Tokyo, Japan) was also loaded on the marker lane indicated. The gel was stained with SYBR Green I Nucleic Acid Gel Stain (TAKARA Bio) and digital images were captured using a GelDoc XR system (Bio-Rad Laboratories). Some DGGE bands were excised, rinsed once with sterile distilled water, and stored at −20°C. A small piece of the gel was directly used as a template for PCR with the same conditions as described above, except that the initial 94°C denaturing step was lengthened to 5 min and the number of cycles was reduced to 34. Each re-amplified product was purified using the QIAquick PCR Purification Kit as described above and directly sequenced using the BigDye Terminator v3.1 Cycle Sequencing Kit (Applied Biosystems, Foster City, CA, USA) with the AMV4.5NF and AMVR primers on an ABI3130 Genetic Analyzer (Applied Biosystems). The sequences of both strands were assembled after removing the primer sequences using DNA Dynamo Sequence Analysis Software (Blue Tractor Software, North Wales, UK). A phylogenetic analysis of DNA sequences was performed using MEGA7 software (16). Prior to the phylogenetic analysis, the band sequences under consideration were aligned together with control sequences obtained from the DDBJ by ClustalW. A phylogenic tree was constructed using the Neighbor-Joining method (30).

### Effects of a competing template on the DDGE analysis

To estimate the sensitivity of the DGGE analysis to contamination, DNA extracts from different fungal strains were mixed and used as PCR templates. The copy numbers of AM 18S rRNA genes in extracts of *Gi. margarita* (MAFF520054), *Rhizophagus intraradices* (MAFF520059), and *Acaulospora longula* (MAFF520060) cultures were quantified by quantitative PCR using the CFX96 Touch^™^ Real-Time PCR Detection System (Bio-Rad) and TB Green Premix Ex Taq II (Tli RNaseH Plus) (TAKARA Bio). The primer set AMV4.5NF and AMVR (see above) was used, and the thermal cycling conditions employed were the same as those described above. A partial fragment of the 18S rRNA gene of *Gi. margarita* (MAFF520054) was amplified with NS1 and NS4 primers (http://nature.berkeley.edu/brunslab/tour/primers.html#18s) using DNA extracted from a single spore as the template. The resulting amplicons were cloned into the pTA2 vector using the TArget Clone TA cloning system (TOYOBO). The constructed plasmid was linearized by digestion with EcoRV and used as a concentration standard in the abovementioned quantitative PCR. The concentration of the templates from three AM fungal cultures were adjusted to 500 copies μL^−1^, and model contaminated templates were prepared by mixing the extracts of target and contaminant species at a ratio of 9:1 (target:contaminant) based on the copy number.

### DNA sequences obtained in the present study

Nucleotide sequence data reported in this manuscript are available at the DDBJ/EMBL/GenBank databases under the accession numbers LC474765–LC474810.

## Results

### DGGE band patterns were useful for the easy identification of AM fungal cultures

Fig. 1A shows the DGGE band patterns of 23 AM fungal cultures belonging to 6 families, 8 genera, and 13 species (Fig. 1B).

**Fig. 2:**
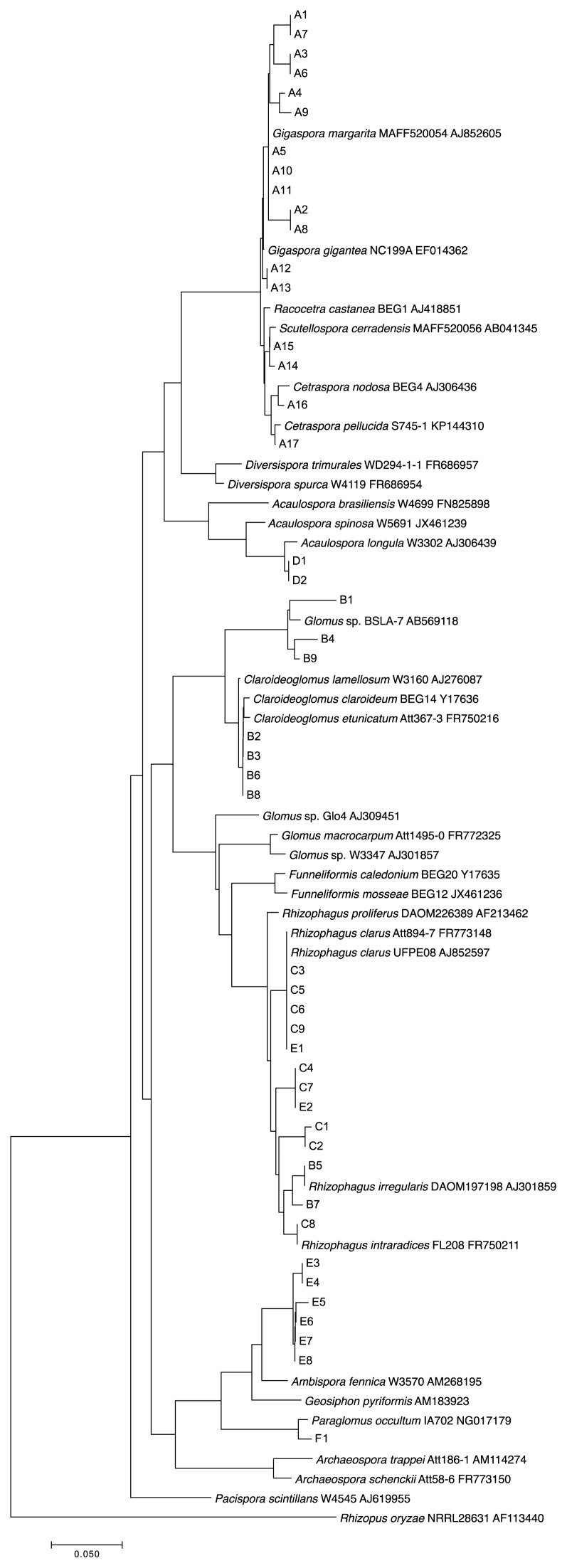
Phylogenic analysis of DGGE bands. The nucleotide sequences of excised DGGE bands (see Fig. 1) were elucidated and the phylogenic tree was constructed. The tree consists of sequences from DGGE bands and AM fungal reference sequences obtained from the DDBJ. The sequence of the non-AM fungus, *Rhizopus oryzae*, was included as an outgroup.

Three *Gigaspora* cultures (two *Gi. margarita* and one *Gi. rosea*) shared a main DGGE band position (designated A5, A11, and A13) with different minor band patterns (lanes 1–3). The phylogenic analysis showed that the sequences of these minor bands belonged to the same clade as the main bands (Fig. 2). Two strains formerly classified as *Scutellospora* (*i.e.*, *Dentiscutata cerradensis* and *Cetraspora pellucida*; lanes 4 and 5, Fig. 1A) showed different patterns with relatively lower positions in the gel (*i.e.*, far from the gel top), as in the case of the three *Gigaspora* cultures. The sequences of their main bands belonged to the same clade as *Gigaspora*, but in a different subgroup. The sequences of minor bands differed from those of the main bands.

Four *Claroideoglomus* cultures (lanes 6–9) shared the same major band position (designated B2, B3, B6, and B8, Fig. 1A), whereas minor band patterns differed from each other. The sequence analysis indicated that some belonged to the *Rhizophagus* clade (B5 and B7, Fig. 1A and 2), suggesting that *C. claroideum* Mu-243 cultures (lane 8) were contaminated with *Rhizophagus* strains. Other minor bands (B1, B4, and B9) were located at positions that were not very close to the main bands.

Four *R. clarus* cultures (lanes 11–13 and 15) showed the same DGGE patterns, consisting of one main band and two minor bands. The DGGE patterns of two *R. intraradices* cultures (lanes 10 and 14) differed from each other and from that of *R. clarus*. The band patterns of two *Acaulospora* species (lanes 16 and 17) were the same, and the sequences derived from their bands were identical (Fig. 2).

The normal PCR reaction (*i.e.*, single-step PCR) used in the present study yielded very few or no products with a template derived from *Ambispora* and *Paraglomus* cultures with one exception (MAFF520055, line 18 in Fig. 1A). The DGGE pattern of the amplicon obtained with single-step PCR was similar to that of *R. clarus* (lanes 11–13 and 15, Fig. 1A), and the sequences of their bands belonged to the *Rhizophagus* clade (Fig. 2), indicating that this culture was contaminated with *Rhizophagus*. Other *Ambispora* and *Paraglomus* cultures that yielded little or no amplification with normal PCR were successfully amplified with two-step PCR. The DGGE patterns of one *Am. leptoticha* (lane 19) and three *Am. callosa* cultures (lanes 20–22) were similar. The DGGE pattern of *P. occultum* was different from those of other strains (lane 23).

The phylogenic analysis of these DGGE bands mostly yielded the expected results, except for some contaminated cultures described above (Fig. 2). *Gigaspora*, *Dentiscutata*, and *Cetraspora* formed one clade; *Acaulospora*, *Ambispora*, *Paraglomus*, *Claroideoglomus*, and *Rhizophagus* were separated into another distinct clade.

### DGGE patterns from single spores

The results shown in Fig. 1 do not clearly demonstrate whether the culture of *C. claroideum* MI-1 (MAFF520092, lane 9) was pure or contaminated with other strains because the pattern contained several bands belonging to a different subclade in the phylogenetic tree (Fig. 2). We have data from another *C. claroideum* culture (not from the NARO Genebank) showing a similar DGGE band pattern as strain MI-1 (data not shown). Furthermore, the DGGE patterns from single spores of MAFF520092 were identical to that shown in Fig. 1A (Fig. S1), implying that strain MI-1 is not contaminated.

**Fig. 3:**
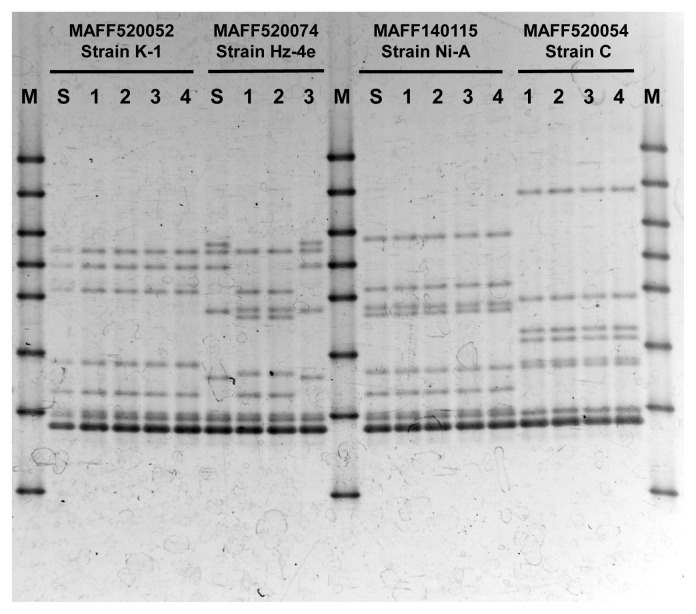
Comparison of soil- and spore-derived *Gigaspora margarita* DGGE patterns. Using different isolates of *Gigaspora margarita*, DGGE patterns obtained from the template extracted from soil (S) or single spores (numbers) were compared. M indicates marker lanes of DGGE Marker IV (Nippon Gene).

Unlike the other species, the DGGE pattern of *Gi. margarita* strains contained many (more than 5) minor bands (lanes 1 and 2, Fig. 1A). To investigate whether this high heterogeneity in the 18S rRNA gene sequence is a general characteristic of this species, the DGGE band pattern of other *Gi. margarita* (MAFF140115 and MAFF520054) were compared. Additionally, to confirm that these minor bands were derived from a single isolate, the DGGE patterns of isolated spores were compared with those of the propagated culture. We were unable to obtain a soil DGGE pattern for MAFF520054 (strain C), presumably because of its low proliferation efficiency. However, the patterns from the different spores were compared, and the results obtained are shown in Fig. 3. Spores isolated from a particular culture generally show the same DGGE pattern, which is identical to the pattern obtained from soil DNA. The only exception is *Gi. margarita* strain Hz-4e (MAFF520074), in which only one out of three spores showed the same DGGE pattern as that of the soil culture.

### The DGGE pattern was sensitive to contamination by different AM fungal cultures

**Fig. 4:**
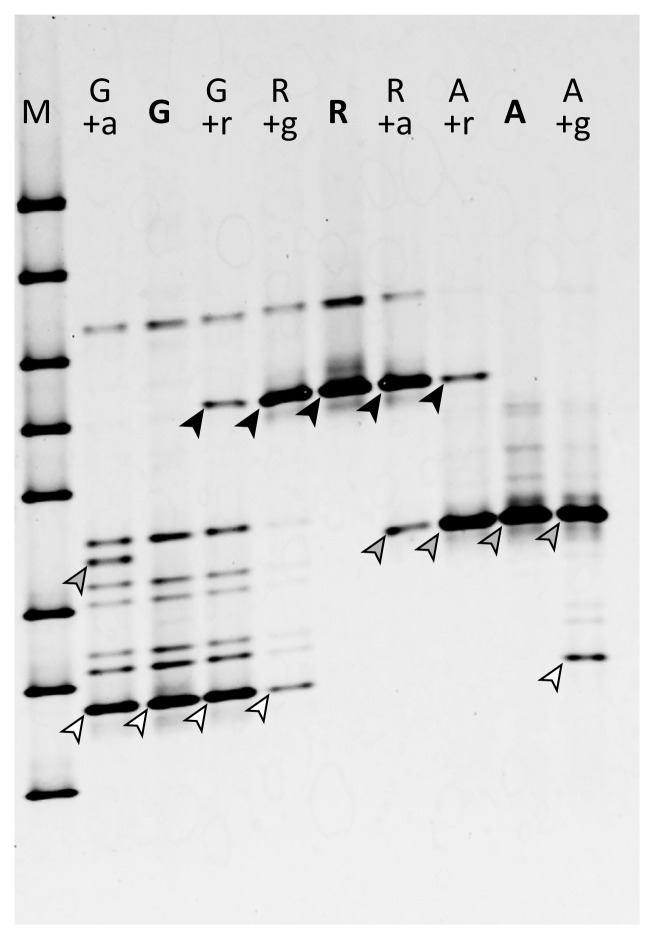
The PCR-DGGE analysis was sensitive to contamination. Lanes marked with a single bold capital letter (G, R, A) contain DNA amplified from the pure AM fungal culture of *Gigaspora margarita*, *Rhizophagus irreguralis*, and *Acaulospora longula*, respectively. Lanes marked with a capital letter plus small letters indicate DNA amplified using a contaminated template, in which the capital letter indicates the main species and the small letter indicates the contaminant (for example, “G+a” means that *Gi. margarita* DNA contaminated with *A. longula* DNA was used as a template). M is a marker lane. The main bands derived from *Gi. margarita*, *R. irreguralis*, and *A. longula* were marked with white, black, and gray arrowheads, respectively.

To elucidate whether contamination by other AM fungal cultures was detectable by the DGGE analysis, the DNA extracts of different cultures were mixed and used as a template for PCR. As shown in Fig. 4, *Gi. margarita* (MAFF520054), *R. intraradices* (MAFF520059), and *Ac. longula* (MAFF520060) were detectable when their DNA contaminated the DNA of other strains at a copy number ratio of 9:1.

## Discussion

As shown in Fig. 1, the DGGE band pattern of each AM fungal strain reflected their DNA sequence-based phylogenetic positions. Therefore, PCR-DGGE allows for the simple evaluation of the status or purity of a given culture. In cases in which the DGGE band pattern is difficult to interpret or ambiguous, it is still possible to confirm the phylogenetic position of the strain in question based on its nucleotide sequence derived from a band excised from the DGGE gel. PCR-DGGE is a more easily applied method than the conventional AM fungal identification method based on spore morphology because it does not require spore isolation and the DNA template may be directly extracted from pot culture soil. In cases in which different strains have proliferated as a mixture in a single pot culture, the DGGE pattern becomes a mixture of each strain. Our experiment showed that when one part of a contaminant strain was mixed with 9 parts of a target strain (in terms of rRNA gene copy numbers), the contaminant strain was detectable. Consequently, PCR-DGGE is a useful and easily applied quality control tool for AM fungal culture collections, which require maintaining the purity of the proliferated strain.

Previous studies that applied PCR-DGGE to analyze AM fungal communities (18, 19, 22) used the primer set NS31-AM1 reported by Helgason *et al.* (12); however, this primer set fails to amplify the rRNA genes of *Archaeosporaceae* and *Paraglomaceae* (28). Sato *et al.* (31) developed a new primer set, AMV4.5NF–AMDGR, which amplifies 18S rRNA genes from a broad range of AM fungal strains including *Claroideoglomus*, *Rhizophagus*, *Gigaspora*, and *Ambispora*. However, Morimoto *et al.* (24) reported that AMV4.5NF–AMDGR amplified sequences from some non-AM fungal species, and this observation has since been confirmed by a pyrosequencing analysis (41), leading to the development of the revised primer set AMV4.5NF–AMVR (which was used in the present study) to reduce non-targeted amplification. Since the region we amplified was nearly the same as that amplified by Sato *et al.* (31) (AMVR was designed to anneal adjacent to the AMDGR annealing site [24]), the DGGE patterns obtained in the present study are very similar to those reported by Sato *et al.* (31).

The DGGE patterns generated in the present study showed that most AM fungal strains produced several minor bands in addition to the main band. Phylogenetically, the nucleotide sequence of these minor bands in most cases belonged to the same clade as that of the main band, indicating intra-isolate variations in ribotypes. It is generally accepted that the rRNA gene sequences of AM fungi show extensive heterogeneity, even if DNA is derived from a single spore (9, 10, 14, 20, 27). Maeda *et al.* (23) recently reported the distinct characteristics of the rRNA genes of AM fungi; highly heterogeneous, no tandem repeat structure, and lower copy numbers in the genome than other eukaryotes. Although they mainly found sequence polymorphisms in 28S rRNA genes and in ITS regions in their target strain *Rhizophagus irreguralis*, this unusual characteristic may explain the heterogeneity of the 18S rRNA gene observed in the present study. The PCR-DGGE analysis provides a clear and easy evaluation of the degree of sequence heterogeneity of the amplified fragment, *i.e.*, how many different sequences are included in a single organism and which sequence is the most abundant. This is another merit of PCR-DGGE over conventional methods, such as cloning-sequencing, which requires the analysis of many clones to estimate the degree of heterogeneity.

The presence of minor bands (and the sequences they represent) in soil DGGE patterns may raise the question as to whether the target strain actually contains these sequences or if they are derived from a contaminating strain. In this case, the DGGE pattern of the single spore needs to be compared with that obtained from soil. If the band pattern of the proliferated strain is consistent with that of the single spore, the minor bands obtained from the soil are not due to contamination, but have instead arisen from the various ribotypes representing a particular proliferated culture. In the present study, the DGGE patterns obtained from proliferated cultures were identical to those from isolated spores with a single exception, indicating that in all but one case, all minor bands were derived from a single strain. However, in the case of the *Gi. margarita* strain Hz-4e (MAFF520074), only one out of three spores showed the same DGGE pattern as the culture. We examined 4 additional spores and found that the DGGE patterns from these spores also differed from that of the soil culture (data not shown). Moreover, the DGGE band patterns of the 6 spores that were different from that of the soil culture were themselves identical with each other, *i.e.*, the pattern was spore-specific. The co-isolation of more than one strain in this culture or contamination with other *Gigaspora* strains are unlikely possibilities because the strain was established via a single-spore isolation procedure in 2003, and we have no other *Gigaspora* strains in our culture collection showing the same DGGE pattern as the “spore-specific pattern”. It is important to note that the source of *Gi. margarita* strain Hz-4e (MAFF520074) is a culture that was propagated in 2016; however, spores were extracted from a culture that proliferated in 2015. The same culture was used as the inoculum source to prepare the 2016 culture. Therefore, the present result indicates that the majority of the spores in the 2015 culture failed to proliferate in 2016. The failure to extract spores from the 2016 culture in order to confirm the status of the soil culture may be a reflection of the low spore formation rate. A better understanding of the present results may be obtained by analyzing spores from a 2017 culture that was propagated from the 2015 culture with a relatively longer propagation period or by conducting a germination assay of spores isolated from the 2015 culture.

By comparing DGGE patterns between soil and spores, we confirmed that different *Gi. margarita* isolates have unique minor band patterns, and these minor band patterns were effectively distinguished among different strains. De Souza *et al.* (10) reported that a greater degree of intraspecies ribotype variation occurs in *Gigaspora*; however, it is important to note that they examined a different position in the 18S rRNA gene to that in the present study. When DNAs from two different lines of *Gi. margarita* were mixed together and then used as a template, the resulting DGGE pattern showed a mixture of patterns from these strains (data not shown). These results suggest that the analysis of DGGE patterns is applicable for tracing specific isolates in the environment as well as elucidating competition or crossing among different *Gi. margarita* isolates.

The recent development of second- and third-generation sequencing technology (21, 26, 41) has reduced the use of PCR-DGGE, particularly for a complex ecological analysis. Nevertheless, PCR-DGGE has its advantages, such its relatively low cost, and since its data output is highly visual, it has the capacity to compare multiple samples loaded on a single gel in a relatively intuitive manner. The method also enables the degree of heterogeneity of a target region to be quickly assessed. Based on these advantages, we conclude that PCR-DGGE is still useful for analyzing flora consisting of a limited number of members. The quality control of proliferated strains in an AM fungal culture collection is one situation in which the merits of PCR-DGGE may be fully utilized.

## SUPPLEMENTARY MATERIAL
