# Supplementary material for: PCR-denaturing Gradient Gel Electrophoresis as a Simple Identification Tool of Arbuscular Mycorrhizal Fungal Isolates
Source: Microbes Environ. 2019 Dec 27;34(4):356–62. doi: 10.1264/jsme2.ME19074 (PMC6934388; doi:10.1264/jsme2.ME19074)
Supplement: Supplementary file 1 [file 34_356_s1.pdf]

Ohtomo, Fig S1

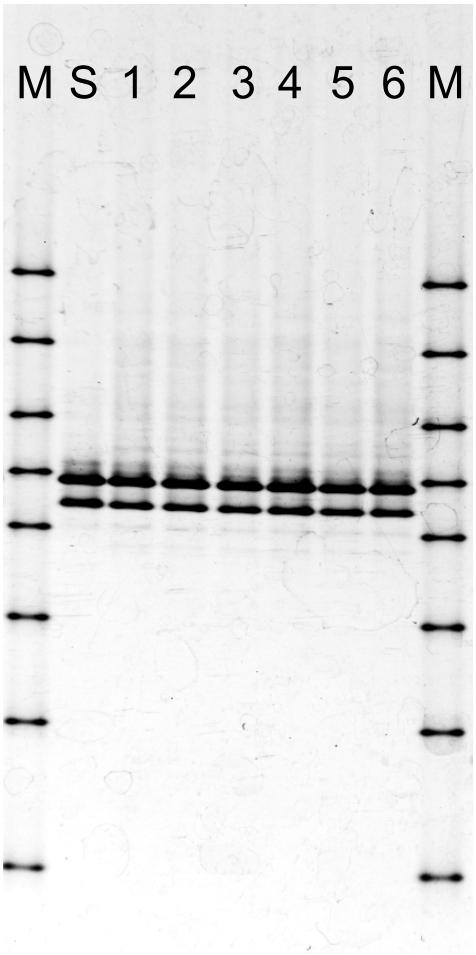

Fig. S1 Comparison of soil- and spore-derived *Claroideoglomus* DGGE patterns

DGGE pattern of AM fungal culture (soil–sand mixture containing AM fungal spores, hyphae and small root pieces of host plants) of *Claroideoglomus claroideum* MI-1 (MAFF520092) were compared with those of isolated spores. S, DGGE pattern of the culture soil, which is the same sample as that loaded in lane 9 in Fig. 1A; 1–6, DGGE patterns of isolated single spores; M, DGGE marker IV from Nippon Gene.
